# Physical Links: defining and detecting inter-chain entanglement

**DOI:** 10.1038/s41598-017-01200-w

**Published:** 2017-04-25

**Authors:** Michele Caraglio, Cristian Micheletti, Enzo Orlandini

**Affiliations:** 1 Dipartimento di Fisica e Astronomia Università di Padova and sezione CNISM, Padova, Italy; 2 SISSA, International School for Advanced Studies, Trieste, Italy; 3 Dipartimento di Fisica e Astronomia Università di Padova and sezione INFN, Padova, Italy

## Abstract

Fluctuating filaments, from densely-packed biopolymers to defect lines in structured fluids, are prone to become interlaced and form intricate architectures. Understanding the ensuing mechanical and relaxation properties depends critically on being able to capture such entanglement in quantitative terms. So far, this has been an elusive challenge. Here we introduce the first general characterization of non-ephemeral forms of entanglement in linear curves by introducing novel descriptors that extend topological measures of linking from close to open curves. We thus establish the concept of physical links. This general method is applied to diverse contexts: equilibrated ring polymers, mechanically-stretched links and concentrated solutions of linear chains. The abundance, complexity and space distribution of their physical links gives access to a whole new layer of understanding of such systems and open new perspectives for others, such as reconnection events and topological simplification in dissipative fields and defect lines.

## Introduction

Mutual entanglement of fluctuating filaments is ubiquitous in nature. In physical contexts, entangled filaments can be created as defect lines in colloidal liquid crystals^1, 2^, turning them into metamaterials with unconventional and tunable physical properties^3, 4^. Linked optical vortices can be formed in propagating laser beams as singularities of the wave’s phase^5^. In chemistry, the syntesis of interlocked molecules is one of the most actively-sought topics^6–8^, with potential applications in the design of molecular machinery with directed molecular motion, complex functionality, novel forms of chirality and self-assembling capabilities^9–12^. Finally, in biology, circular interlocked DNA structures or catenanes arise as physically inevitable byproducts of replication and recombination^13–15^.

Mathematically, the topological entanglement of two curves is defined only if they are both closed. In such case, their linked state can be detected, and its complexity quantified, with suitable topological invariants^16^.

Open curves, being topologically unlinked, are by definition beyond the scope and reach of such invariants. Therefore no measure is currently available for capturing their degree of mutual intricacy nor for locating where the entanglement actually is on the given curves.

**Figure 1:**
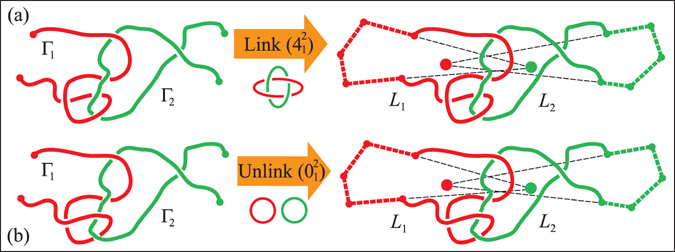
Detection of physical links: The sketches illustrate a key step of the procedure, namely how a pair of open chains can be turned into rings by closing each of them with an auxiliary arc (thick dashed lines) pointing away from the centre of mass of the partner chain (filled circles). To minimize the additional entanglement that may arise during closure, the segments pointing away from the centre of mass of the other chain are typically chosen to be much longer than the radius of gyration of the chains. This closure “at infinity” turnes the two chains, Γ_1_ and Γ_2_, into the components, *L*_1_ and *L*_2_, of a proper link, whose topology can be established with suitable invariants, such as the multivariate Alexander polynomial. The procedure correctly distinguishes between the non-trivial entanglement in panel (a), a Solomon physical link, from the unlink of panel (b). The two link types are indicated, respectively, as ${4}_{1}^{2}$ and ${0}_{1}^{2}$ in the Rolfsen’s notation^16^.

Yet, a simple definition inspired by a common sense notion of physical entanglement between chains (see Fig. 1a,b) is needed in countless contexts. In response to this necessity various *ad hoc* approaches and observables have been introduced in to describe the intertwining of polymers in a melt^17–19^, of peptide chains in protein complexes^20, 21^.

Here, we tackle this unsolved problem by providing an intuitive, robust and efficient method to close pairs of open chains and use the resulting topological states to define the *physical links* formed by the curves (see Fig. 1). The method is general, straightforward to implement, and hence applicable to the diverse physical situations where multi-chains interactions are relevant. It has the further advantage of being applicable to *any* pair of chains, be they open or closed, to identify their linked portions.

The notion of physical link, therefore, allows for detecting multi-chain entanglement both at the global level, through the type and complexity of the physical link, and at the local one, by pinpointing the chain portions where it resides.

In the following we first introduce the concept of physical links and the key algorithmic steps of the method used to define it. We next illustrate its application in different contexts of general interest. Specifically, we first discuss two linked rings that are pulled apart mechanically, then move to the scaling analysis of the physically entangled region in equilibrated links, and finally address the challenging and debated case of concentrated solutions, or melts, of linear chains.

## Results

### Physical links

To illustrate the idea of physical links let us consider the two pairs of chains shown in Fig. 1a,b. Since the chains are open, both instances are not topological links, and their nominal geometrical complexity is equivalent, because they both present 4 inter-chain crossings when viewed from most angles. Yet, their entanglement is readily perceived as being qualitatively, or physically different. In fact, pulling the chains in opposite directions - a case discussed below -results in a further tightening of the entanglement in (a), while it would disengage and separate the chains in (b).

These differences, which are elusive to topological or geometrical measures, can actually be detected and formalised with the procedure described in Fig. 1(a,b). The essential step of the method is to turn a physical link into a proper topological one by suitably closing each chain. Several possible closure schemes can be envisaged. In fact, various ones have been adopted for closing single chains and establish their physical knotted state^22–27^. Here, because the added closing arcs should minimally interfere with the chain entanglement, we close each chain “at infinity” with an auxiliary arc that points away from the centre of mass of the other chain, see Fig. 1(a,b). After such step, the original spatial mutual entanglement of the open chains in panels (a) and (b) of Fig. 1 is appropriately captured as a Solomon link and an unlink, respectively. This simple procedure therefore provides an intuitively correct assignment of the physical linking state of the original pair of chains.

### Linked portions of open or closed curves

**Figure 2:**
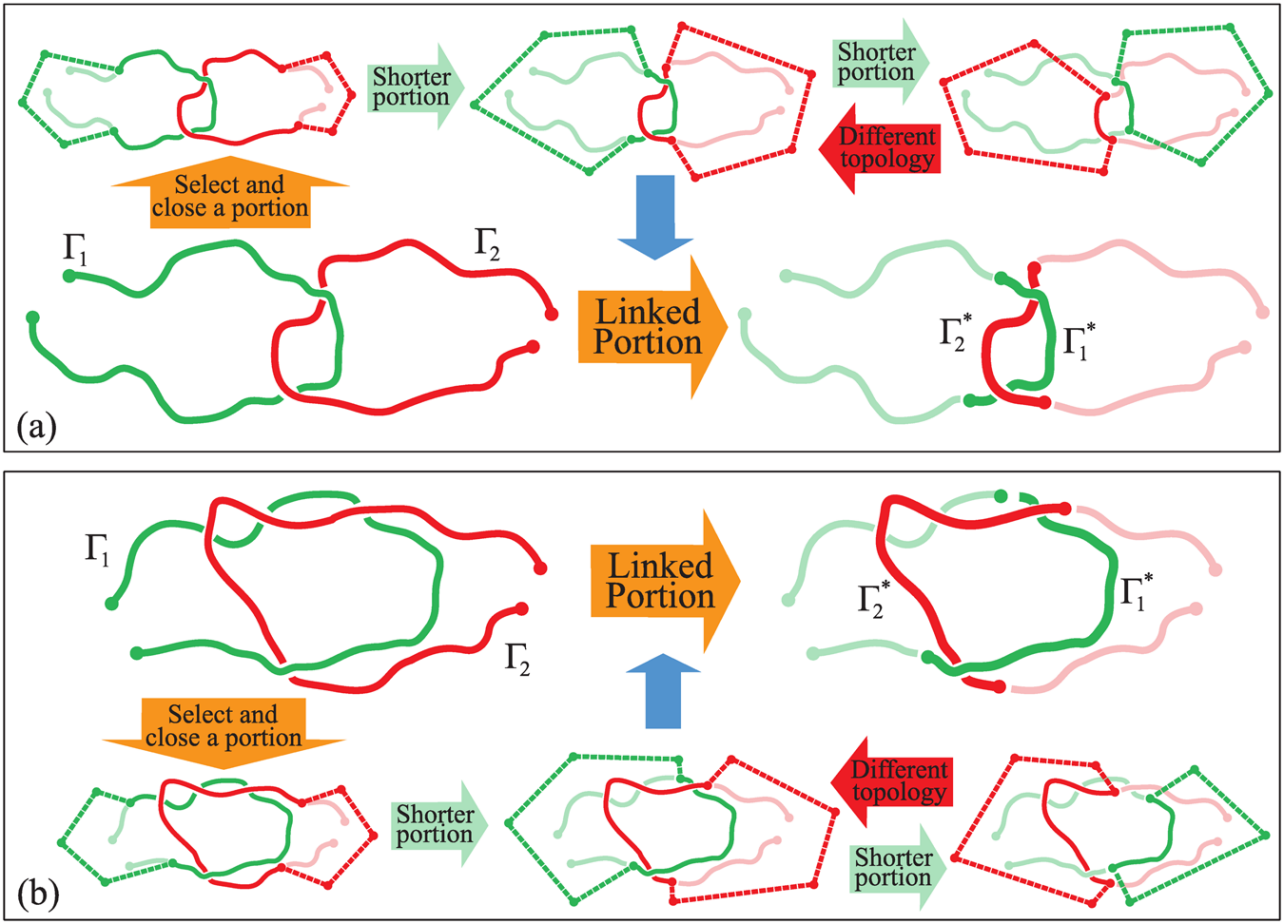
Linked portions of open or closed curves: The linked region of two curves, Γ_1_ and Γ_2_ is defined as the shortest portion that, upon closure, has the same topology of the original physical link. For open curves, such region can be found by a stochastic top-down reduction of the chains from their termini, as illustrated here. The two panels pertain to physical links with the same topology, the Hopf or ${2}_{1}^{2}$ link, but with large differences for the length of their linked portion, which is indicated with a * subscript in the right part of the panels. For closed chains, the contour reduction can be started from randomly chosen points on the rings, see also Figs S1 and S2 in SI.

The notion of physical links can be seamlessly used to locate the portions of the two chains where their physical entanglement resides. The required steps are sketched in Fig. 2(a,b), which portrays two physical links that are identical by type (Hopf physical link) but are otherwise clearly different for the extension of their linked portions.

To locate the latter, one starts by considering the whole ensemble of pairs of sub-arcs from the two chains. Next, one retains only the pairs of arcs that have the same physical link type as the entire chains. Of these pairs, the one with the smallest total arc-length is the sought physically-linked portion of the original chains.

This search scheme is applicable to both open and closed curves. If fact, it can detect the entangled region of two chains of irrespective whether they establish a physical link, or a topological one.

As it is shown in Fig. 2, the search strategy correctly returns regions of very different total arc-length for the examples of Fig. 2(a,b).

In practical contexts, the identification of the physically-linked portion requires a two-tier approach for sifting through the combinatorial space of the sub-arcs while computing the corresponding link types (after closure). Effective strategies for performing either steps are presented in the methods section and in Figs S1 and S2 of the Supplementary Information, SI).

### Tensile mechanics of links

The mechanical pulling of two concatenated rings is an ideal system for applying these concepts and validate them, too. At sufficiently high stretching forces, in fact, the physical entanglement is easily revealed and located by simple visual inspection.

Although such systems are nowadays experimentally accessible to molecular design and micromanipulation techniques, the effects of topology on the elastic response of catenanes is still largely unexplored. We show that much physical insight into this relationship can be gleaned by applying the notion of physical links and, in particular, of linked portions.

In our setup we consider four different link types: the Hopf link (${2}_{1}^{2}$), the Solomon link (${4}_{1}^{2}$), the Whitehead link (${5}_{1}^{2}$) and the Star of David link (${6}_{1}^{2}$). The rings, which are modelled as semi-flexible circular chains of *N* = 120 beads each in canonical equilibrium at temperature *T*, are pulled in opposite directions by a constant force (see Methods for details).

**Figure 3:**
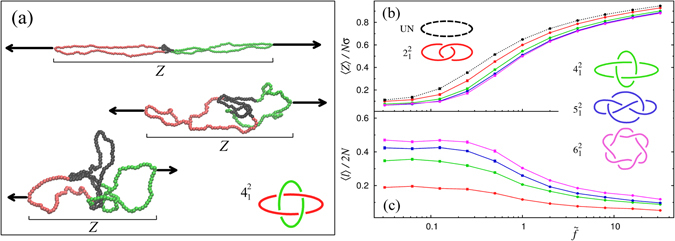
Stretching topologically linked loops: (**a**) Configurations of linked pairs of loops (Solomon link) in canonical equilibrium at temperature *T* at three values of the stretching force, *f*. Each loop is modelled as a semiflexible ring of *N* = 120 beads and persistence length *lp* = 5*σ* where *σ* is the bead diameter. The linked portions are highligted in black and *Z* is the span projected in the stretching direction, which cannot be larger than half the total contour length, *N*. (**b**) Average normalized span, 〈*Z*〉/*Nσ*, as a function of the reduced force $\tilde{f}=f\sigma /{k}_{B}T$. The solid curves refer to different link types (see labels) while the dashed one is for a single loop twice longer (2*N* = 240). (**c**) Force dependence of the average contour length of the linked portions for the linked loops of (**b**).

Typical configurations of the Solomon link at different stretching forces are given in Fig. 3a. The link configuration at high force is clearly elongated in the stretching direction, which is taken as the *z* Cartesian axis. Its physically-linked portion, highlighted in black, is tight and matches the region that one would pick from visual inspection. The same high force causes an analogous tightening for the other types of links although, as the link complexity increases the length of the chain used up to maintain the topological constraint, i.e. the linked portion, increases too, see Fig. S2 in SI. As shown in the other panels of Fig. 3a, when the force is reduced, the rings relax and the physically-linked region expands concomitantly.

The force-extension profiles for the links are given in Fig. 3b. The curves portray the relative average span of the links measured along the pulling direction, 〈*Z*〉/*N*, as a function of the adimensional (reduced) force strength, $\tilde{f}$. For reference, the relative span of a single ring of equivalent length (2*N*) is shown too.

The spread of the force-extension curves in Fig. 3b gives a vivid illustration of how significantly the tensile response depends on the topology of the catenate. In particular one notes that the stretching compliance varies monotonically with the nominal complexity of the entanglement: It is highest for the single ring and lowest for the star-of-David links. This trend could be expected in the limit of high forces since the extension of the linked rings should be smaller than the “doubled” single ring by an amount about equal to the shortest possible length of the linked portion, which clearly increases with link complexity. Figure 3b shows that this trend holds at all forces and that, surprisingly, the topology-dependent variations in extension are largest at intermediate forces.

To better clarify this intriguing behaviour, we profiled the average contour length of the physical link, $\langle \ell \rangle$, as a function of $\tilde{f}$. The curves for the normalised length, $\langle \ell \rangle \mathrm{/2}N$, are given in Fig. 3c. One sees that at large forces, $\tilde{f} > 2$, the chain fraction covered by the physical link is relatively small (<20%), and only weakly dependent on the link type. Instead, for intermediate forces ($0.3 < \tilde{f} < 2$) the linked region varies rapidly with $\tilde{f}$. This arguably reflects the entropy driven swelling of the linked portion that outcompetes the mechanical tightening at these forces, similarly to what happens for tensioned knots^28^. Finally, for small forces, $\tilde{f} < 0.3$, the linked region is delocalized over a significant fraction of the rings (as much as 50% for the Star of David link). The delocalization somewhat loosens the geometry-topology coupling and, consequently, the extension has a weaker dependence on the link-type.

The non-monotonic force-dependence of the spread of the extension curves in Fig. 3b is clearly a notable feature from the point of view of polymer physics and may be of applicative relevance too. In fact, it suggests that stretching curves, nowadays experimentally accessible by micromanipulation or microfluidic techniques^29, 30^, could be used as a topological fingerprints. This rich and unexpected phenomenology cannot be unveiled without the concepts and tools introduced here.

### Scaling properties of linked portions

**Figure 4:**
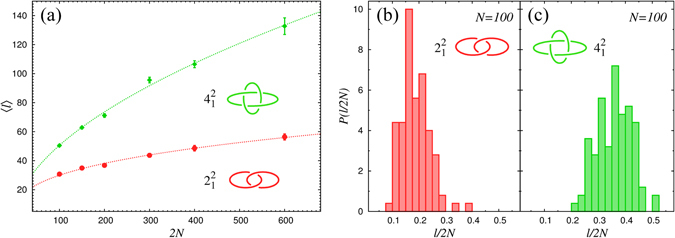
Scaling and statistical properties of linked portions: (**a**) Average contour length of the linked portions, $\langle \ell \rangle$, as a function of the total contour length 2*N*, for Hopf (red circles) and Solomon (green diamonds) links formed by semi-flexible rings each of *N* = 100 beads. For each set of data the solid curve corresponds to power law fits (see Eq. 1). Panels (b) and (c) Probability distribution of the fraction of contour length in the linked portion, $\ell \mathrm{/2}N$, for Hopf (**b**) and Solomon (**c**) links.

As a further application, we examine how the average contour length of the linked region, $\langle \ell \rangle$, depends on the chain length, *N*, at zero force. The $\langle \ell \rangle$ versus *N* curves for Hopf and Solomon link are given in Fig. 4a.

The data points are well interpolated by the dotted lines that correspond to power scaling laws,

1 $$\langle \ell \rangle \sim {N}^{\alpha }.$$

with *α* = 0.36 ± 0.05 and *α* = 0.54 ± 0.05 respectively for the Hopf and Solomon link. The fact that in both cases *α* is well below 1, is a strong indication that, even discounting finite size corrections, the average length of the linked region grows sublinearly with *N*.

This implies that the average inter-chain entanglement of long rings is weakly localised. This is reminescent of the weak localization observed for intra-chain entanglement, i.e. knots^26, 28, 31, 32^. The two cases, however, present qualitative differences in the length distribution of the entangled portion. In fact, the modal length of the linked portions appears to increase with chain length (see Fig. S5 in SI), while the modal knot length does not^32–34^. More importantly, at fixed *N*, the shape of the distribution $P(\ell )$ depends qualitatively on the link type being broader for more complex links (see Fig. 4b and Fig. S5 in SI).

### Physical links and polymer melts

We now turn to a paradigmatic case for inter-chain entanglement, namely a concentrated solution of linear chains. This is a classic, and yet still open problem in polymer theory, with important practical ramifications^19^ and implications for biological systems^35^.

**Figure 5:**
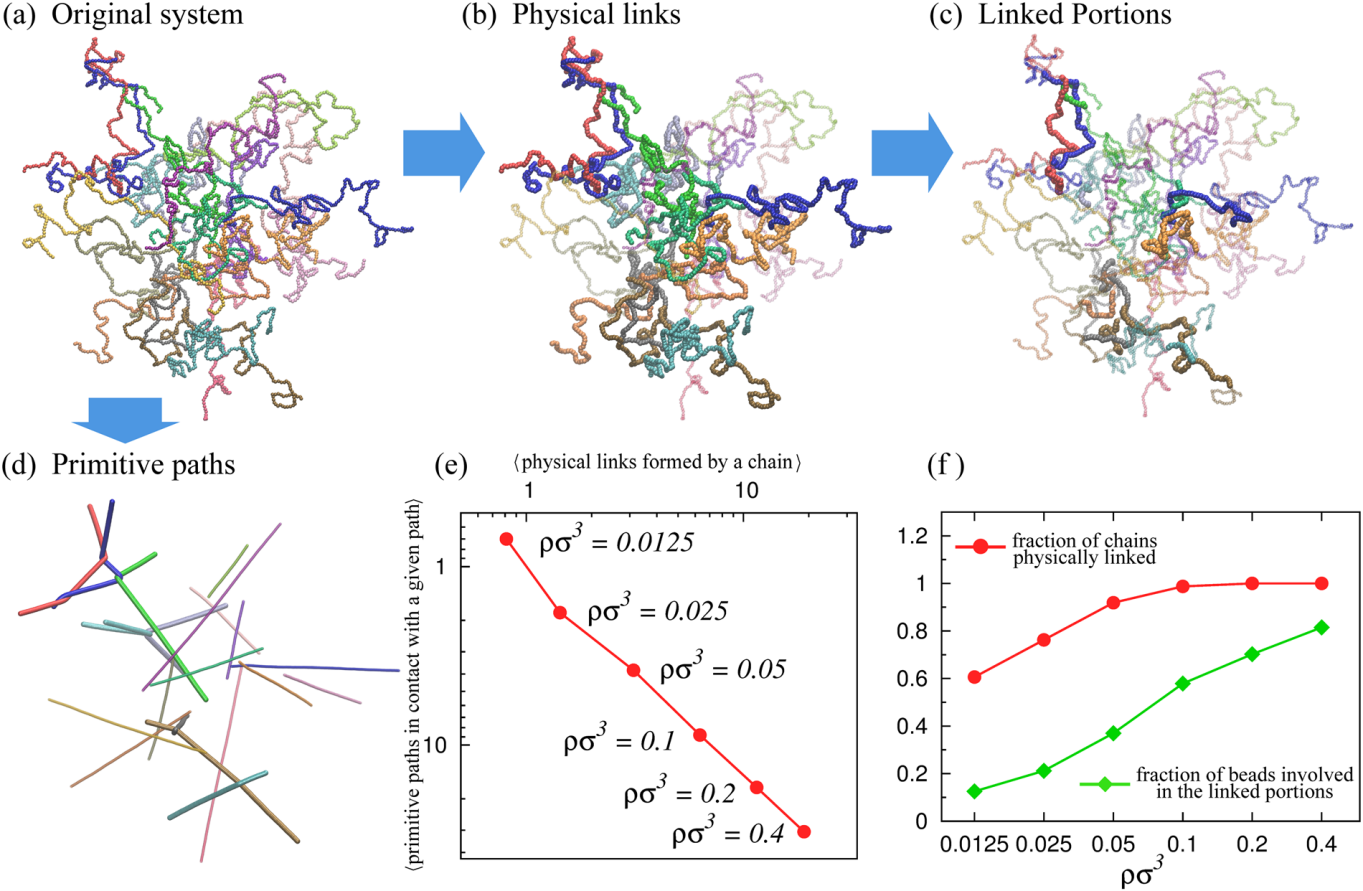
Physical links in melts: (**a**) Equilibrated configuration of a solution of 20 semiflexible linear chains (*l*_*p*_ = 5*σ*) each with 250 beads of diameter *σ*. The system has a monomer concentration *ρσ* ^3^ = 0.0125. (**b**) Topological characterisation of configuration (**a**) made in terms of physical links (brighter and thicker chains). For comparison the corresponding primitive path representation is given in panel (d) where paths with at least one kink are made thicker. In panel (d) only the linked portions of the physical links in (**b**) are highligthed. Note that, unlike the primitive path representation (**d**) the one based on either physical links (**b**) or linked portions (**c**) resides in the original configurational space. (**e**) Quantitative comparison, at different solution density, betwen description (**b**,**d**) made in terms of the average number of physical links experienced by a chain vs the average number of primitive paths in contacts with a given path. (**f**) Fraction of chains that are physically linked (red circles) and fraction of beads involved in the linked portions (green diamonds).

Here we consider concentrated dispersions of hundreds of semi-flexible chains (*l*_*p*_ = 5*σ*), each composed by hundreds of beads. A typical configuration, or microstate, is shown Fig. 5a which shows an ensemble of 20 chains of 250 beads at the monomer density *ρσ* ^3^ = 0.0125.

The characterization of such systems is typically based either on global invariants from knot theory^36–38^ or on primitive path analysis and entanglement length^17–19, 39–41^. The latter concepts have, indeed, proved particularly valuable to rationalize in quantitatively reliable terms, the ensuing viscoelastic, rheological and relaxation properties^19, 42, 43^.

We recall that the primitive paths of a microstate are obtained by contracting the contours of the chains while keeping their termini fixed and without allowing strand passages. When the chains are disentangled, the contracted paths are essentially straight segments and their contour lengths closely matches the distance of the chain endpoints. However, for a genuinely intricate microstate, each primitive path will typically be interlocked with several contacts with other paths, forming hooked kinks. In this case, the contour length of the primitive paths will be appreciably longer than their end-to-end distances.

The latter point is illustrated in Fig. 5d which shows the primitive paths of the microstate in panel a, which is picked at the smallest considered density, *ρσ* ^3^ ~ 0.0125. Those paths that feature one or more kinks are shown with a thicker trace. One notes that at this relatively small density, the kink or interlocking points involve only two chains at a time, consistent with earlier findings^19, 44^. Yet, individual chains can be entangled with more than one partner chain, see e.g. the triplet of interlocked chains in the upper left part of panel d. At the same density, *ρσ* ^3^ ~ 0.0125, about 60% of the filaments are entangled with one or more other ones; this percentage grows rapidly with the solution density and saturates already at *ρσ* ^3^ ~ 0.1, see panel f.

As we now discuss, the valuable insight offered by primitive path analysis can be aptly complemented by the concepts of physical links and linked portions, which give access to an additional layer of quantitative profiling.

As a first step we queried all chain pairs and, using the detection scheme of Fig. 1, identified those that were physically linked, see thicker traces in Fig. 5b.

The good correspondence of the physically linked chains and the interlocked primitive paths is well evident from the visual inspection of panels (b) and (d). From a more quantitative point of view, the consistency of the two strategies to single out inter-chain entanglement is shown in Fig. 5e. This plot shows, for various system densities, the ensemble averages of the number of interlocked primitive paths versus the number of physical links that a chain forms pairwise with the other chains. The correlation of the two quantities is excellent and shows, *a posteriori* the robustness of the local entanglement detection with either strategies. In this respect it would be interesting to use the strategy based on physical links to estimate the entanglement length, a goal that will require a more systematic study of these systems at various, and larger, polymer contour lengths.

The additional, novel element that is brought about by the present analysis is the identification of the physically-linked portions, which are captured in their original (uncontracted) positions along the chains and in the embedding three-dimensional space.

For the microstate of interest, the linked portions are highlighted in Fig. 5c. The image provides a vivid representation of how local entanglement is distributed in space, and particularly, for the fact that they appear to be organised in small clusters, a feature that, to our knowledge, has not been pointed out before. At the same density of the microstate in panel (a) the linked portions cover, on average, about 12% of the contour length of the chains. This percentage steadily grows with the system density and retains a good dynamic range even when other conventional order parameters, such as the percentage of physically linked chains, have saturated already, see Fig. 5f.

### Topological spectrum of polymer melts

**Figure 6:**
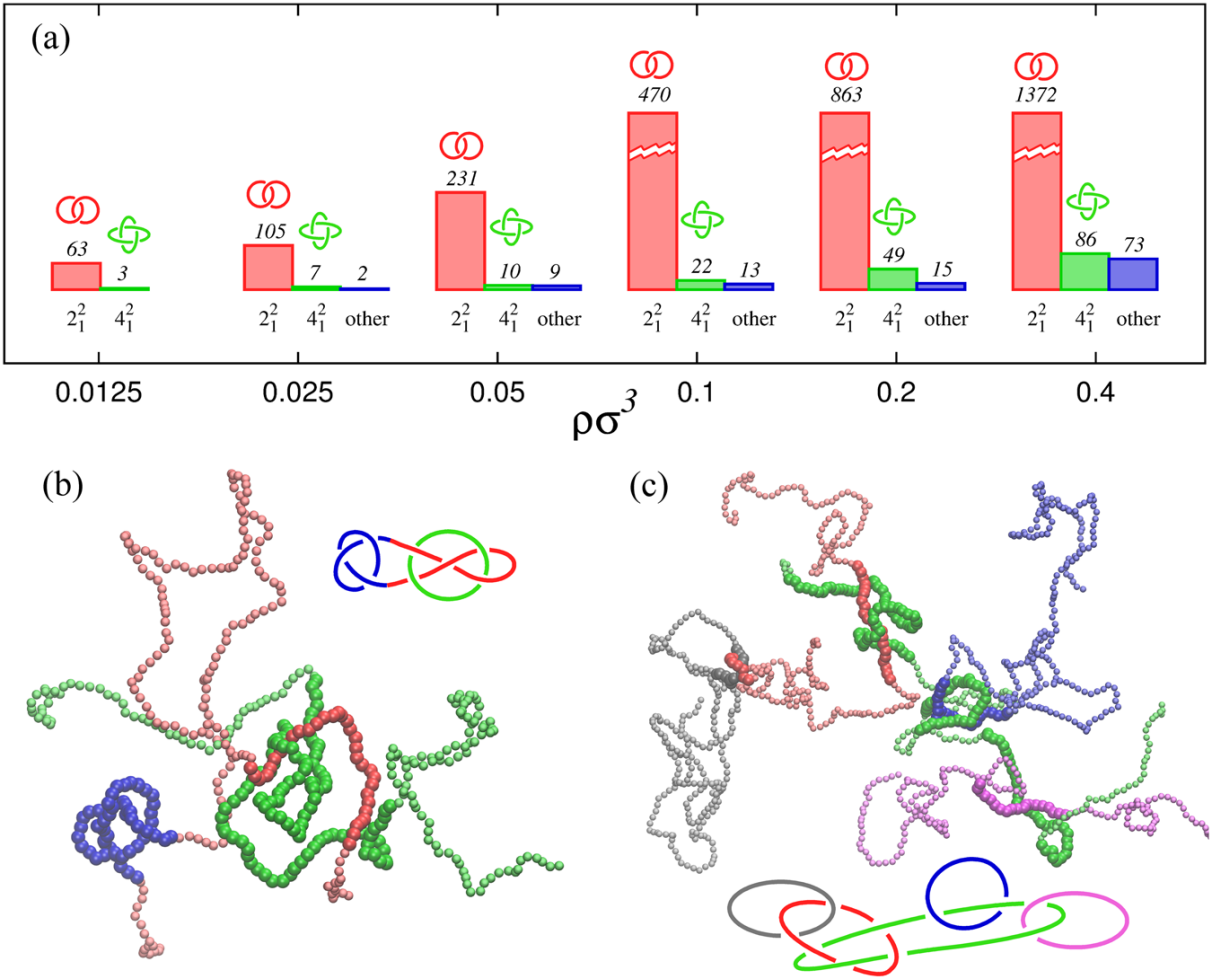
Topological complexity in melts: (**a**) Topological spectrum associated to the physical links found in the melt at different solution density *ρσ* ^3^. As *ρ* increases, the category “other”, where all the more complex links are collected, gets more and more populated. Two configurations of this category, found at *ρσ* ^3^ = 0.1, are represented in panels (b,c). Panel (b) refers to a physical Whitehead link that additionally host a physical knoy (trefoil). Panel (c) represents a 5 component physical link where the chains are catenated in pairs with either Hopf or Solomon links.

An even more relevant description of the entanglement complexity of the solution can be obtained, within our general framework, by looking at the *topological spectrum of the melt*, that is the relative abundance of the various topological types observed in the physical links. This is reported in Fig. 6a for different solution densities.

For sufficiently diluted solutions the spectrum consists mostly of Hopf physical links with a minor presence of Solomon rings. These are the first two topologies in the complexity ladder of the Rolfsen’s table^16^ of links. In fact, one observes the most common links at all considered densities are the Solomon and Hopf types, the latter being more probable by several times.

Moving to the less common physical links we note the occurrence of the Whitehead links, see Fig. S6, which is particularly notable because it is the simplest non-trivial link that is not homologically linked (i.e. has linking number zero)^16^. This indicates that, for an accurate and exhaustive topological profiling of physical links in a polymer melt, one must necessarily resort to invariants, such as the multivariate Alexander determinant, that are more sophisticated than the linking number.

Besides the Whitehead case, the more complex links include the Star-of-David topology as well as exotic links such as those visualised in Fig. 6(b,c) (see also movies M1 and M2 in SI). They represent two paradigmatic examples of link families: one (panel b) is a physical Whitehead-like link where one component is also tied in a trefoil, or 3_1_ knot, while the other (panel c) is a 5-component link made by 3 Hopf links and one Solomon link.

As the contour length of the chain increases, the complexity of their physical linking will increase too. In particular, homotopical links should progressively outnumber homological ones^45^ (i.e. links with *Lk* ≠ 0) and the inevitable chain overlaps should eventually result in giant multicomponent links. In such clusters many filaments of the system are linked either by direct physical interlocking or indirectly, via the concatenation of few mediating chains. Small-scale example of both cases are shown in Fig. 6c and Fig. S7d in SI.

A systematic search for multicomponent links can be straightforwardly accomplished by taking suitable powers of the Boolean matrix, *M*, whose general entry *M*_*ij*_, is equal to 0 or 1 depending on whether the chains *i* and *j* are linked or not. The entries of the *k*-th power of *M* that are equal to 1 will correspond to chain pairs that are linked via *k*−1 mediating filaments.

By applying this search scheme one establishes that already at *ρσ* ^3^ = 0.1, the system displays practically a single giant multi-component physical link. In fact, Fig. S7c in SI shows that almost any chain in the system is physically linked to any other via at most 4 intermediate chains. For *ρσ* ^3^ = 0.4 any two given chains are typically linked via as few as 2 intermediate chains, see Fig. S7b in SI.

One caveat regarding the aforementioned multicomponent-link search scheme is that it is inherently dependent on the detection of the pairwise linking of their components. As a consequence, Brunnian links, which include the well-known Borromean links, cannot be detected^16^. These more general instances could be addressed by higher-order topological invariants^16, 46^ or, also, by a mechanical pulling procedure that could extract all topologically linked chains after they have been closed. To do that, one would need to adopt more generalised closure schemes than the one discussed here, that is inherently pairwise in its formulation.

### Dynamics and reconnection of physical links: perspectives

So far we focussed on the equilibrium or static properties of entanglement filaments and rings. The dynamical evolution of entanglement is, however, a source of fascinating phenomenology for various complex physical systems.

Besides polymer melts, where one can monitor the temporal development of de Gennes’ tubes created by topological constraints^18, 19^, the most promising applications are arguably for systems where the entangled string-like objects are free from physical connectivity constraints. Such systems include defect lines in liquid crystals^2, 47^, flux lines in fluids^48^ and superfluids^49, 50^ and optical vortex lines^5, 51, 52^. It is precisely for the lack of a physically-bonded backbone, that these entangled defect or vortex lines can evolve, anneal and break up in dramatic ways.

Characterizing such systems as physical links could provide an unprecedented insight into their dynamical behaviour by allowing for monitoring the size, position (both in space and along the lines contour) and complexity of the entangled portions. We envisage that this would lead to better understanding of the intriguing phenomenology of reconnection events of dissipative flux and vortex lines and of the conservation laws that typically underpin them^53^.

## Conclusions

To summarize, we have introduced a novel approach to detect and measure the entanglement between open chains that relies on the notions of physical links and of linked portions. The viability and usefulness of these concepts was illustrated in different prototypic contexts for multi-chain entanglement. These were: the elastic properties of topologically-linked loops under stretching, the scaling behavior of the linked portion of equilibrated links, and the spatial arrangement of linear polymers in a melt. Among the results obtained in these examples we cite the scaling behaviour of the portion of catenanes involved in the physical links and a full characterisation of the melt entanglement established in terms of a topological link spectrum and spatial distribution of the linked portions. This definition of mutual entanglement has the remarkable advantage of being very simple to implement and general enough to be applied to disparate contexts where the entanglement between fluctuating filaments is relevant, including the reconnection events and topological simplification recently reported for dissipative fields and defect lines^2, 5, 47–50^.

## Methods

### Model and simulation setup

Filaments are modelled as chains of *N* beads of mass *m* and diameter *σ*. The potential energy of each chain includes the following three contribution which account respectively for the chain connectivity^54^, the excluded volume interaction and the chain bending rigidity

2 $$\begin{array}{rcl}{U}_{{\rm{FENE}}} & = & -\sum _{i}^{N-1}15\epsilon {(\frac{{R}_{0}}{\sigma })}^{2}\,\mathrm{ln}[1-{(\frac{{d}_{i,i+1}}{{R}_{0}})}^{2}],\\ {U}_{{\rm{bend}}} & = & \sum _{i=2}^{N-1}K(1-\frac{{\vec{b}}_{i-1}\cdot {\vec{b}}_{i}}{|{\vec{b}}_{i-1}|\,|{\vec{b}}_{i}|}),\\ \,\,{U}_{{\rm{LJ}}} & = & \sum _{i,j > i}^{N}4\epsilon [{(\frac{\sigma }{{d}_{i,j}})}^{12}-{(\frac{\sigma }{{d}_{i,j}})}^{6}+\frac{1}{4}]\theta {\mathrm{(2}}^{\mathrm{1/6}}\sigma -{d}_{i,j}),\end{array}$$

where ${d}_{i,j}=|{\vec{r}}_{i}-{\vec{r}}_{j}|$ (${\vec{r}}_{i}$ denotes the position of the *i*-th bead) and ${\vec{b}}_{i}\equiv {\vec{r}}_{i+1}-{\vec{r}}_{i}$ is the *i*-th chain bond. The parameter *R*_0_ = 1.5*σ* is the maximum bond length, *θ* is the Heaviside function and $\epsilon$ is the characteristic unit of energy that we set equal to *k*_*B*_ *T*. By setting *K* = 5.51$\epsilon$, we simulate semiflexible chains with persistence length *l*_*p*_ = 5*σ*. This choice is dictated by the need of simulating a sufficiently realistic model of a semiflexible chains at varius contour lengths and to keep the computational costs of link identification at a manageable level. Simulations of linked loops under tensile forces have been performed with the constant force pulling protocol implemented as follows: for a given initial linked configuration we choose the bead with the smallest *z*-coordinate. This left-most bead, that identifies one loop (say loop 1) is kept fixed in space. We then apply a force *f* along the positive *z* direction to the right-most bead of loop 2. Denoting by *L* the distance, along the *z* coordinate, of the two beads, this amount to add a potential energy, *U*_pull_ = −*fL*. Given the total potential energy *U* = *U*_FENE_ + *U*_bend_ + *U*_LJ_ + *U*_pull_, the dynamic of the *i*-th bead is described by the Langevin equation: $m{\ddot{\vec{r}}}_{i}=-\xi {\dot{\vec{r}}}_{i}-\nabla U+\vec{\zeta }$ where *ξ* is the friction coefficient and $\vec{\zeta }$ is the stochastic delta correlated noise. The variance of each Cartesian component of the noise, ${\sigma }_{\zeta }^{2}$ satisfies the fluctuation dissipation relationship ${\sigma }_{\zeta }^{2}=2\xi {k}_{B}T$.

As customary^54^, we set $m/\xi ={\tau }_{LJ}$ with ${\tau }_{LJ}=\sigma \sqrt{m/\epsilon }=\sigma \sqrt{m/{k}_{B}T}$ and the Brownian time ${\tau }_{Br}=\sigma /{D}_{b}$ with ${D}_{b}={k}_{B}T/\xi$ the characteristic simulation time step. In all the simulations of linked loops the initial configuration is a pair of unknotted rings interlocked by hand with the desired link type. The Langevin equation of motion are integrated numerically with the LAMMPS package by using standard values for the friction coefficient, beads mass and with time step ${\rm{\Delta }}t=0.05{\tau }_{LJ}\sim 0.4$ *ns* ^55^.

For each link type, we generate 100 trajectories in which the force is progressively diminished from its maximum value to zero by discrete amounts. At each value of the force the dynamics is followed for 10^7^ timesteps, thereby covering a time period of 3.7 ms. This time has been estimated to be sufficiently long to achieve an equilibrated system at a given force.

For the melt problem we consider *M* semiflexible (*l*_*p*_ = 5*σ*) linear chains with *N* = 250 beads. Equilibrated samples of such systems were generated at different monomer density *ρσ* ^3^ by changing either *M* or the simulation box size and with the stochastic scheme of ref. 56.

### Detection of physical links

The detection of a physical link requires first the transformation of each chain into a loop. The closure procedure is sketched in Fig. 1 where it is applied to two different physical links ((a) and (b)): given two open chains Γ_1_ and Γ_2_ (left panel) we first compute their centre of mass. We then draw two segments starting from the extremities of Γ_1_ and pointing away from the centre of mass of Γ_2_. The transformation of Γ_1_ into a loop is then performed by joining the ends of these segment along the surface of a sphere with radius very large compared to the extension of the pair (red dashed arc). This closure at “infinity” is repeated for Γ_2_. The final outcome is a pair of loops (*L*_1_, *L*_2_) whose topology *τ* is determined by computing the two-variables Alexander polynomial^16, 57^.

### Locating the linked portion

Given a pair of either open or closed chains (Γ_1_, Γ_2_) with link *τ* the estimate of the linked portion, (${{\rm{\Gamma }}}_{1}^{\ast }$, ${{\rm{\Gamma }}}_{2}^{\ast }$), is obtained by looking for the shortest physical link whose topological link, computed upon closure, is compatible with *τ* (see Fig. 2). To determine the physical link of all possible pairs of subchains (*γ*_1_, *γ*_2_) included in (Γ_1_, Γ_2_) can be very computationally demanding and here we adopt a top-down searching scheme based on a bisection method. Moreover, to avoid the detection of physical links in regions of (Γ_1_, Γ_2_) that at iteration *k*−1 (i.e. more coarse grained) were not found compatible with the topological target *τ*, a self-learning proceure is implemented. This decreases substantially the set of potential pairs (*γ*_1_, *γ*_2_) to be examined at iteration *k*. If at iteration *k* a pair (*γ*_1_, *γ*_2_) is physically linked with topology *τ* while no pairs with the same topology are found at the next (more refined) iteration *k* + 1, the procedure stops and (*γ*_1_, *γ*_2_) is identified with $({{\rm{\Gamma }}}_{1}^{\ast },{{\rm{\Gamma }}}_{2}^{\ast })$ (see red arrow in Fig. 2). The flow chart of the algorithm is drawn in Figs S1 and S2 of SI and detailed in their figure caption.

## Electronic supplementary material

Supplementary material for
