# Supplementary material for: Physical Links: defining and detecting inter-chain entanglement
Source: Sci Rep. 2017 Apr 25;7:1156. doi: 10.1038/s41598-017-01200-w (PMC5430864; doi:10.1038/s41598-017-01200-w)
Supplement: Supplementary file 1 — Supplementary material for [file 41598_2017_1200_MOESM1_ESM.pdf]

Supplementary material for "Physical Links:defining and detecting inter chain entanglement"

Michele Caraglio*

Dipartimento di Fisica e Astronomia Università di Padova
and sezione INFN, Via Marzolo 8, I-35131 Padova, Italy

Cristian Micheletti†

SISSA, International School for Advanced Studies, via Bonomea 265, I-34136 Trieste, Italy

Enzo Orlandini‡

Dipartimento di Fisica e Astronomia and Sezione INFN,
Università di Padova, Via Marzolo 8, I-35131 Padova, Italy

(Dated: March 18, 2017)

PACS numbers: 36.20.Ey, 02.10.Kn, 82.35.Lr, 87.15.A-

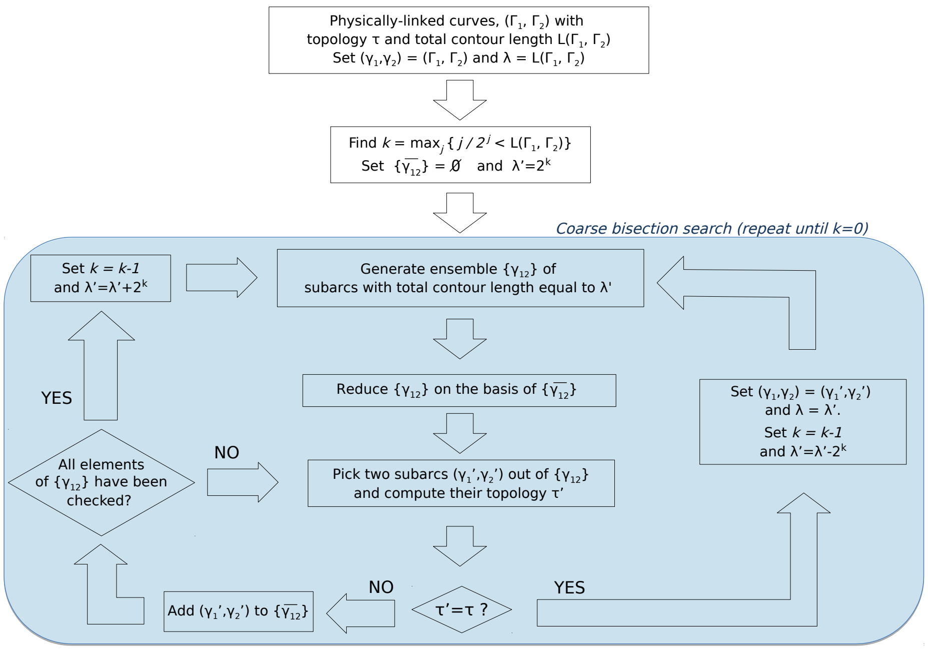

Figure S S1. The linked region of two (physically) linked curves, (Γ_1, Γ_2) with topology τ and contour length $L(\Gamma_1, \Gamma_2)$ is found with a repetitive stochastic top-down approach. The idea is to extract, from (Γ_1, Γ_2), pairs of sub-arcs (γ_1', γ_2') with length λ' and to retain only those having link type τ. Among these, the one with the smallest length is identified as the best approximation of the linked portion. The scheme is based on a top-down search that starts from the set $\{\gamma_{12}\} = \{(\gamma_1', \gamma_2')\}$, with length λ'. Initially $\lambda' = 2^k$ with $k = \max_j\{j \mid 2^j < L(\Gamma_1, \Gamma_2)\}$ while in next iterations $k \to k-1$ and $\lambda' \to \lambda' \pm 2^k$ according to a bisection rule. Not all the pairs in $\{\gamma_{12}\}$ are worth to be analysed and to save computational cost we consider a self-learning procedure that makes use of the subset $\{\overline{\gamma_{12}}\}$ generated at the previous level of iteration (see below) to keep only those that may contain the link type τ. At each iteration step, if a pair (γ_1', γ_2') has link type $\tau' = \tau$, it is chosen as the temporary linked portion of (Γ_1, Γ_2) at that step, we set $k \to k-1$, $\lambda' \to \lambda' - 2^k$ and move to the next iteration step. If this is not the case, the pair $\{(\gamma_1', \gamma_2')\}$ is stored in the subset $\{\overline{\gamma_{12}}\}$ for the self-learning procedure. If no pair in $\{\gamma_{12}\}$ has τ' compatible with τ, we set $k \to k-1$, $\lambda' \to \lambda' + 2^k$ and move to the next iteration step. The iteration procedure stops when $k = 0$. The cost of the computational intensive step involving the consistency check of topologies τ' and τ can be substantially reduced by following the hierarchical scheme shown in Figure S2.

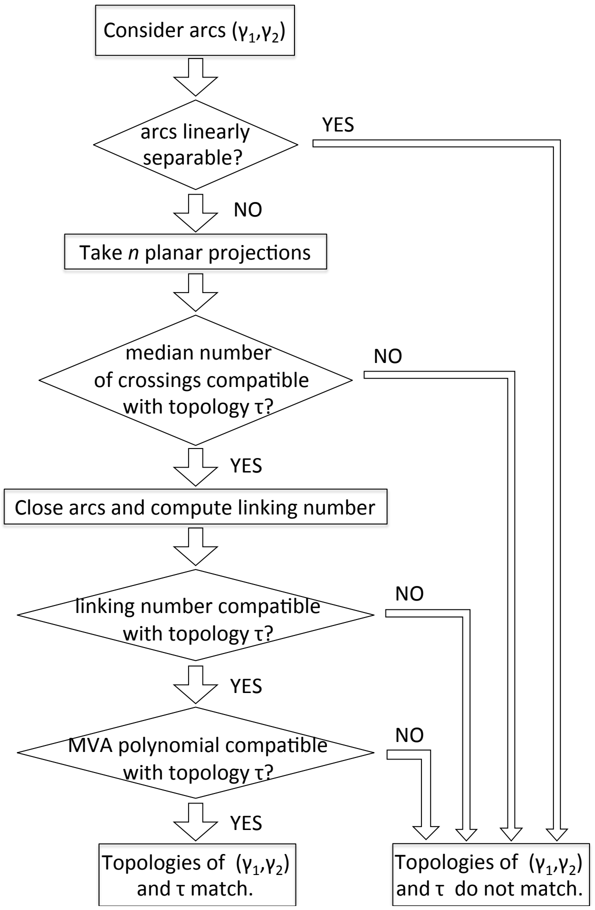

Figure S S2. The consistency check of topologies tau' and τ is best performed by going through a series of checkpoints of increasing computational cost. First one checks if the subarcs are not separable by a plane (otherwise they are clearly unlinked). Then one takes a certain odd number, n, of planar projections (in our cases $n = 13$) and checks that in the majority of projections the number of inter-arc crossings is not smaller than required by the target topology, τ. Next, after closure of the arcs, it is checked that the linking number of the arcs is compatible with the target topology and, if so, the multi-variate alexander polynomials are finally computed ad compared.

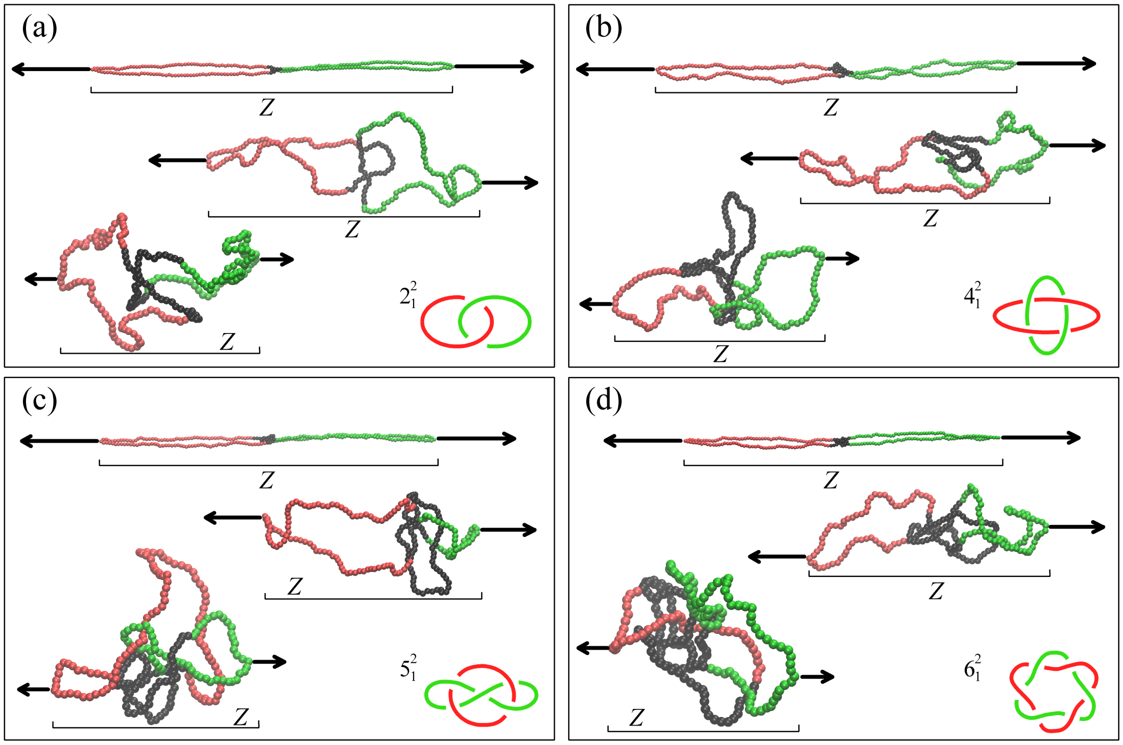

Figure S S3. Steady state configurations of Hopf (a), Solomon (b), Whitehead (c) and Star of David (d) links under stretching. In each panel the snapshots refer to three different values of the reduced extensile force $\tilde{f} = f\sigma/k_BT$: $\tilde{f} = 8, 1$ and 0.125. Z denotes the extension of the two loops along the pulling direction. For each configuration the linked portion, measured with the algorithm illustrated in Fig.S1 and Fig.S2, is highlighted in black. It is apparent that, for weak forces, more complex links occupies larger portion of the loops and the corresponding configurations are more compact.

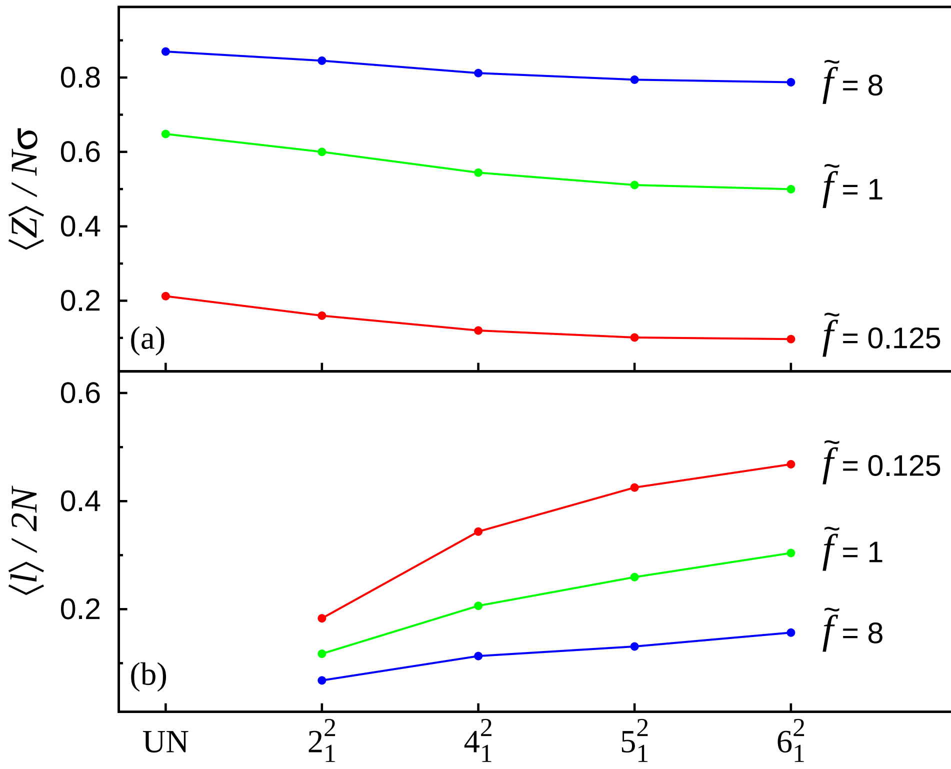

Figure S S4. (a) Average extension Z of linked loops and average size of the linked portions ℓ (b) as a function of link complexity. The total contour length is $2N = 200$ Different curves refer to different value of the reduced force $\tilde{f} = f\sigma/k_BT$ (see legend). The averages are taken over 100 independent trajectories. As qualitatively shown in Figure S3, at fixed $\tilde{f}$ more complex links are less extended. Moreover they require a longer portion of the loops to be accomodated. For istance at $\tilde{f} = 0.125$ roughly half of the loops are needed to accomodate a star of David link.

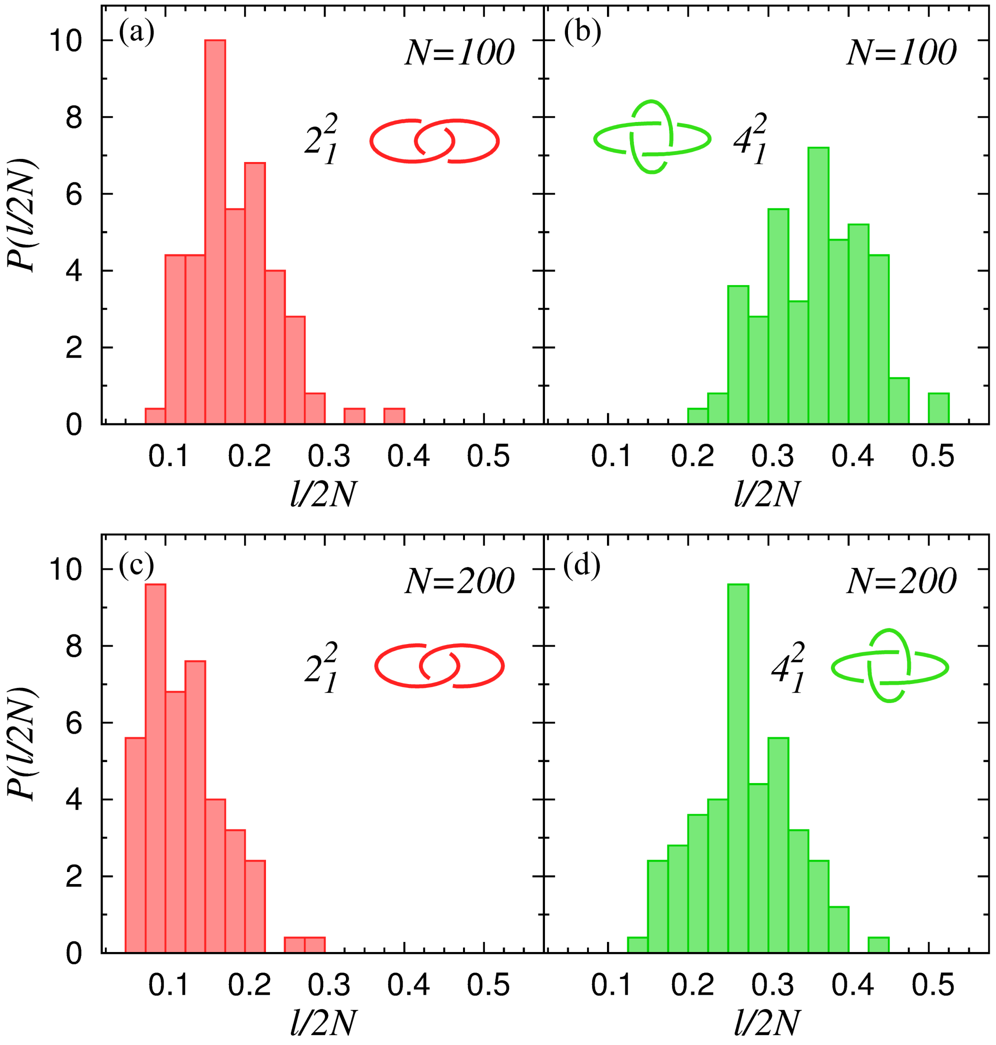

Figure S S5. Probability distribution of the fraction of contour length involved in the linked portion $\ell/2N$, for the Hopf link (a,c) and Solomon link (b,d). Each component of the link is a ring of either $N = 200$ (a,b) or $N = 100$ (c,d) beads. Each distribution has been estimated by analysing 100 independent configurations. One can notice that more complex links are characterised by a broader distribution in the length of the linked portions. Moreover, at fixed link type, the modal value of the distributions depends on N.

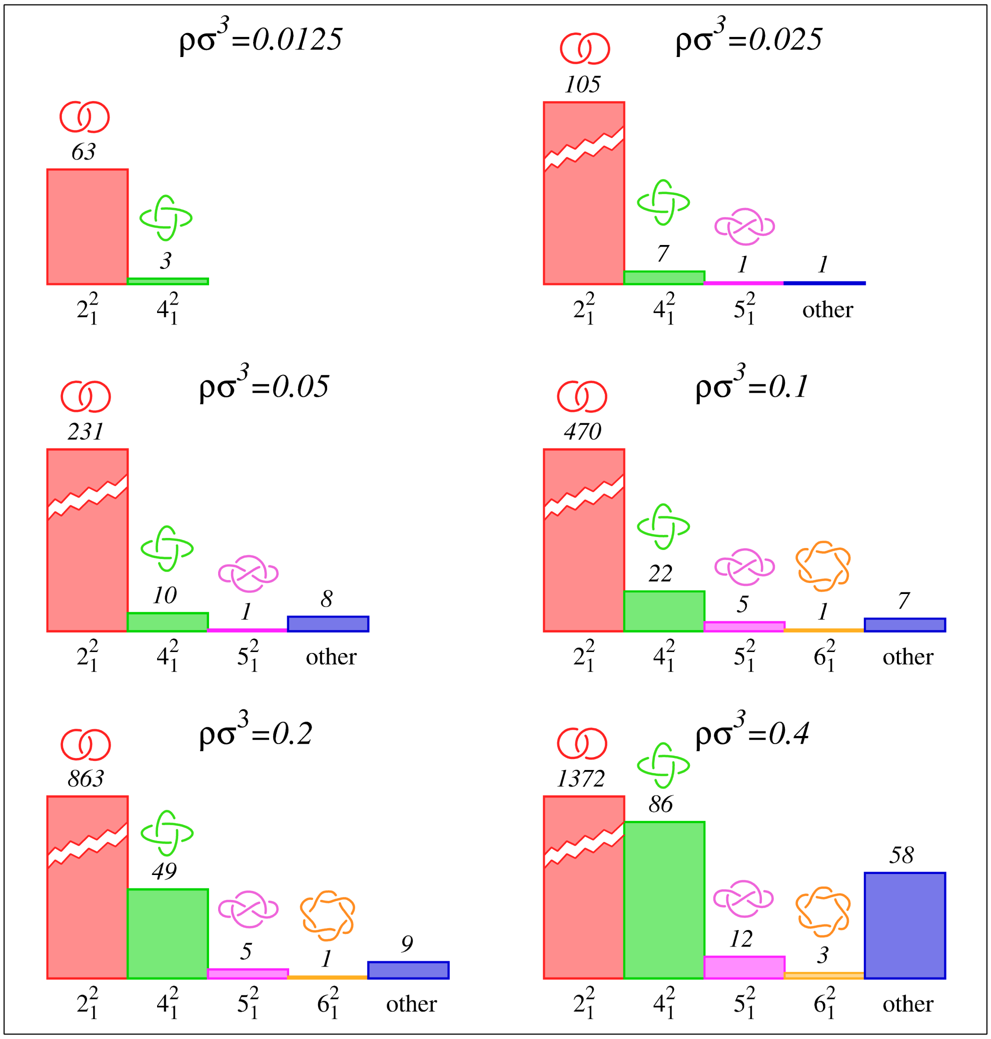

Figure S S6. Detailed topological spectrum of a system of linear polymers estimated at different values of solution density ρ . This is determined by computing the topological link type associated to each physical link detected in a configuration. Note that, as ρ increases (from 0.0125 up to 0.4) physical links with more complex topology are observed. The presence of the Whitehead link that has trivial linking mumber is quite remarkable. In the category "other" more exotic links such as the ones presented in Fig. 6 of the main text are collected.

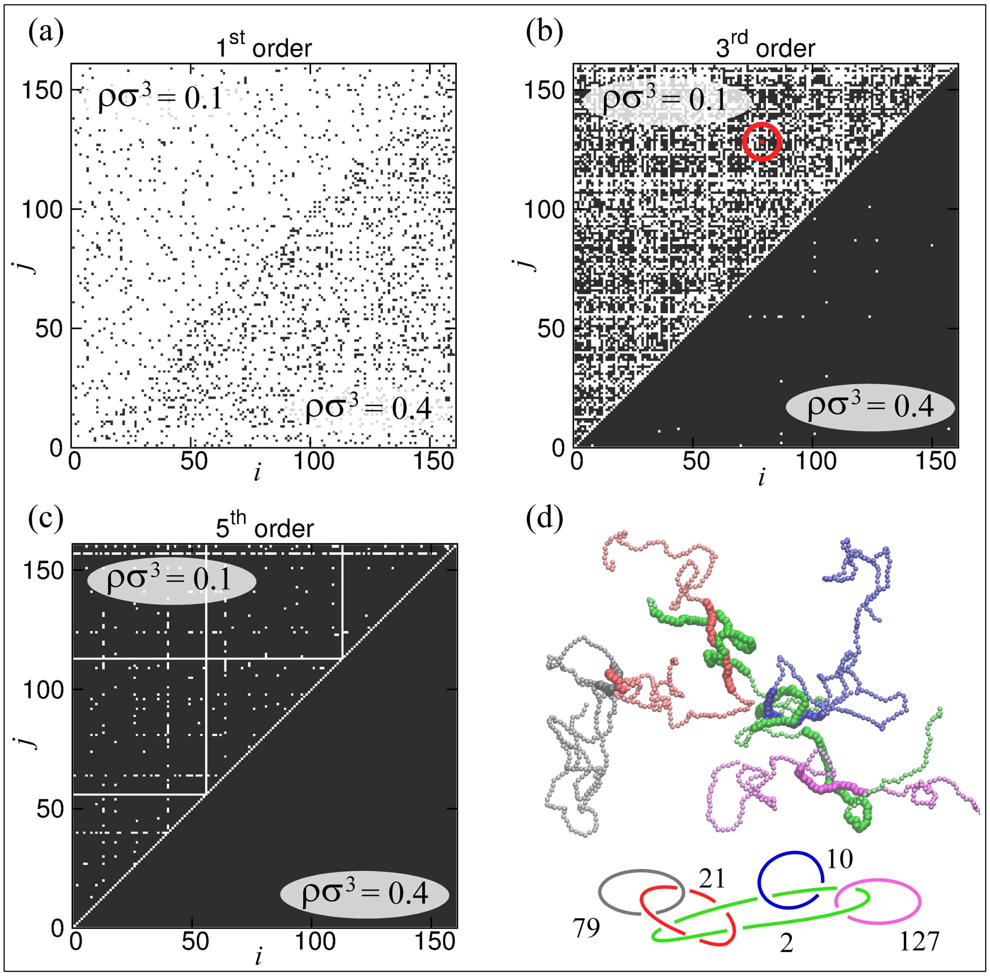

Figure S S7. Examples of the search scheme for multicomponents homotopical links based on pairwise linking. The physically-linked state any two chains in the systems is encoded in a matrix, M whose general entry M_{ij}, is equal to 0 or 1 depending on whether chains i and j are linked or not. The notion of linking is based on the homotopical criterion (i.e the two-variable Alexander polynomial) applies to the two chains after their closure. Panel (a) shows a graphical representation of the matrix (dots represent non-zero entries) for two configurations of the polymer melts discussed in the main text: one with density $\rho\sigma^3 = 0.1$ (upper triangle) and one with density $\rho\sigma^3 = 0.4$ (lower triangle). By taking the suitable powers of this connectivity matrix one can establish whether two chains are indirectly linked via the concatenation of other mediating chains. The matrices in panel (b) and (c) correspond to the third and fifth power of M, and hence their non-zero entries reveal those pairs of chains that are linked by, respectively up to 2 and up to 4 mediating chains. It is seen that these indirect connection effectively span most of the chains at $\rho\sigma^3 = 0.1$ and all of them at $\rho\sigma^3 = 0.4$. An example of a cluster of chains that are direct and indirectly linked to each others is shown in panel (d). The chains are extracted from a larger system of $N = 160$ chains at density $\rho\sigma^3 = 0.1$. The numbers below the rings denote the indexing of the chains in the simulations. Entry $M_{79,127}$ (and its symmetric counterpart) is equal to zero because the two rings are not directly linked (upon closure). The same entry of the second power of M, $M^2_{79,127}$ is zero too, because there is no single ring that bridges them. Instead, as highlighted in red in panel (b), $M^3_{79,127} = 1$ because the two rings are concatened via rings no. 2 and 21.
